# Early Immune Response Elicited by Different *Trypanosoma cruzi* Infective Stages

**DOI:** 10.3389/fcimb.2021.768566

**Published:** 2021-11-25

**Authors:** Brenda Celeste Gutierrez, Estela Lammel, Stella Maris González-Cappa, Carolina Verónica Poncini

**Affiliations:** ^1^ Laboratorio de Inmunología Celular e Inmunopatología de Infecciones, Instituto de Investigaciones en Microbiología y Parasitología Medica (IMPaM), Universidad de Buenos Aires, Consejo Nacional de Investigaciones Científicas y Técnicas (CONICET), Buenos Aires, Argentina; ^2^ Departamento de Microbiología, Parasitología e Inmunología, Facultad de Medicina, Universidad de Buenos Aires, Buenos Aires, Argentina

**Keywords:** *T. cruzi*, parasite stages, dendritic cells, cell activation, T cells

## Abstract

*Trypanosoma cruzi* is a protozoan parasite that affects millions of people in Latin America. Infection occurs by vectorial transmission or by transfusion or transplacental route. Immune events occurring immediately after the parasite entrance are poorly explored. Dendritic cells (DCs) are target for the parasite immune evasion mechanisms. Recently, we have demonstrated that two different populations of DCs display variable activation after interaction with the two infective forms of the parasite: metacyclic or blood trypomastigotes (mTp or bTp) *in vitro*. The skin constitutes a complex network with several populations of antigen-presenting cells. Previously, we have demonstrated *T. cruzi* conditioning the repertoire of cells recruited into the site of infection. In the present work, we observed that mTp and bTp inoculation displayed differences in cell recruitment to the site of infection and in the activation status of APCs in draining lymph nodes and spleen during acute infection. Animals inoculated with mTp exhibited 100% of survival with no detectable parasitemia, in contrast with those injected with bTp that displayed high mortality and high parasite load. Animals infected with mTp and challenged with a lethal dose of bTp 15 days after primary infection showed no mortality and incremented DC activation in secondary lymphoid organs compared with controls injected only with bTp or non-infected mice. These animals also displayed a smaller number of amastigote nests in cardiac tissue and more CD8 T cells than mice infected with bTp. All the results suggest that both Tp infective stages induce an unequal immune response since the beginning of the infection.

## Introduction

*Trypanosoma cruzi* is the etiological agent of Chagas disease, the most important parasitic infection in Latin America. Six to seven million people are infected in endemic areas (WHO, 2021), and as a consequence of active migration, hundreds of thousands of individuals are infected in non-endemic areas such as the United States and Europe (Requena-Méndez et al., 2015; Bern et al., 2019).

The parasite presents different routes of entry. In endemic areas, human infection mainly occurs by vectorial transmission when metacyclic trypomastigotes (mTp), found in insect feces, enter into the host *via* mucous membranes (oral/ocular/gastric) or damaged skin, such as the wound made by the bite of the triatomine insect. In addition, blood trypomastigotes (bTp) enable congenital or transfusion transmission. While congenital transmission occurs in endemic and non-endemic areas, infection by transfusion is described in areas without blood donor screening and testing for Chagas (Bern et al., 2019). Some structural and molecular differences have been described for the two Tp forms that explain, in part, why each form is adapted to infect and/or disseminate through different host microenviroments (Cortez et al., 2012). For example, while bTp are more sensitive to gastric degradation in comparison to mTp (Cortez et al., 2012), the last ones are less resistant to the complement (Yoshida and Araguth, 1987). A previous work has consistently demonstrated the replication of the parasite in the gastric mucosa after oral infection with mTp (Hoft et al., 1996); however, studies involving the skin are scarce.

*Trypanosoma cruzi* acute infection shows high parasitemia and parasite load in tissues followed by a chronic phase where the parasite persists in tissues as amastigote nests (Ward et al., 2020). After a variable period of latency, it was described that 30% of the patients can display symptoms including arrhythmias, heart failure, and sudden death (Andrade and Andrews, 2005; Marin-Neto et al., 2007).

Parasitemia during the acute phase of the infection can be followed by an inflammatory storm that includes different cellular mediators such as microbicide molecules and cytokines. After innate immunity takes place where tissular macrophages and NK cells play central roles in the control of the parasite, adaptive immunity is characterized by polyclonal expansion of T and B cells with a potent effector response that controls the parasite load but is inefficient to eradicate it (Basso, 2013). Parasite persistence is actually associated with multiple evasion mechanisms displayed by the parasite, including cell reclusion in immune-tolerant sites, the development of non-dividing dormant forms, and the active ways in which *T. cruzi* avoids the triggering of the immune responses or exhausts the existing ones (Lewis and Kelly, 2016; Sánchez-Valdéz et al., 2018).

*Trypanosoma cruzi* infection triggers an inflammatory response, effective in the control of the parasite spreading; however, in comparison with other infections, it is delayed in time (Sánchez-Valdéz et al., 2018). Dendritic cells (DCs) link innate and adaptive immunity. In steady state, DCs patrol tissues and are central in the recognition of danger signals. They capture, process, and present antigens to T cells. Basically, DCs are sentinels specialized in the regulation of adaptive immunity not only by priming T-cell responses but also by driving immune tolerance. DC subsets are heterogeneous and display a complex network with specializations related to differentiation/residence niches (Merad et al., 2013; Anselmi et al., 2020).

DCs are present in all tissues and biological barriers and are probably one of the first immune cells to interact with *T. cruzi* when the portal of entry is the skin. Previous reports have shown the tolerogenic properties of bTp on human monocyte (Mo)-derived DCs (Van Overtvelt et al., 1999) or bone-marrow-derived DCs in mice (Poncini et al., 2008). In addition, our group has demonstrated the relevance of early monocyte mobilization at the site of the parasite entry and the relevance of this heterogeneous population, which includes Mo-DCs, at the first steps of infection (Poncini and González Cappa, 2017). More recently, results obtained *in vitro* demonstrated that not only the parasite infective stage but also the origin and activation status would define the subset behavior of DCs (Gutierrez et al., 2020). The objective of the present work is to study the scope of the findings obtained *in vitro* in the experimental model of infection. Our results demonstrate that mTp and bTp infective stages are differentially recognized by the immune system since the beginning of infection. Animals intradermally inoculated with mTp display low parasite load, enhanced DC activation, and benign manifestations of the experimental infection compared with animals injected with bTp. These results suggest that not only the biological properties of the parasite but also the proper activation of the immune response compromises the outcome of *T. cruzi* infection.

## Materials and Methods

### Animals, Parasites, and Infection

C3H/HeN and CF1 mice were maintained at the animal facilities of IMPaM UBA-CONICET, Facultad de Medicina, Universidad de Buenos Aires and bred *ad libitum* under sanitary barrier in specific pathogen-free conditions (Gutierrez et al., 2020).

Briefly, RA *T. cruzi* bTp were obtained from whole blood at the peak of parasitemia of CF1 mice (7 days post-infection) by differential centrifugation as previously reported (Poncini et al., 2008). Epimastigotes (epi) were routinely differentiated from bTp and cultured *in vitro* in liver infusion tryptose (LIT) medium at 27°C to the exponential phase of growth and centrifuged at 3,000×*g* for 15 min at 10°C (Isola et al., 1986). mTp were obtained by one round of differentiation from epi as described by Gutierrez et al. (2020). After *in vitro* culture of 10 × 10^7^ epi in 10% fetal bovine serum (FBS) LIT plus Grace’s insect medium (MERC) and incubated at 27°C in tightly closed culture flasks, parasites were purified in DEAE column equilibrated with PBS-glucose (20%) at pH 8.2. Purity was analyzed by microscopic examination.

Ten- to 12-week-old C3H male mice received intradermic (ear or hindfoot) or intraperitoneal injection with 1,000 parasites. Animal health condition, parasite load, and mortality were periodically recorded.

All experiments were performed according to protocols CD N° 04/2015 approved in Res. 923/21 by the University of Buenos Aires’s Institutional Committee for the Care and Use of Laboratory Animals (CICUAL) in accordance with the Council for International Organizations of Medical Sciences (CIOMS) and International Council for Laboratory Animal Science (ICLAS), international ethical guidelines for biomedical research involving animals.

### Tissues and Cell Samples

For cell collection, histology, or DNA extraction, samples were obtained at different time points after injection with PBS (negative control of infection; NI) or the parasite. Tissue sections (25 mg) were preserved at −80°C until processing for DNA extraction. Epidermal cells were obtained from ear skin sheets using trypsin (1% and 0.3%; Sigma), as previously described (Poncini and González Cappa, 2017). Spleens were enzymatically digested with hyaluronidase type IV-S (200 U/ml; Sigma-Aldrich) and collagenase type II (250 U/ml; Gibco, Invitrogen) cocktail. After mechanical and/or enzymatic disaggregation, samples from the spleen, skin, and draining lymph nodes (dLNs) were homogenized through a tissue strainer and debris removed by passing samples through a 100-µm nylon mesh. When it was necessary, red blood cells were lysed by Tris 0.83% ammonium chloride buffer, pH 7.2, and mononuclear cells were obtained by centrifugation (400×*g*, 30 min) in Histopaque 1083 (Sigma) gradient. Cells were washed in fresh medium and viable cell count was determined by Trypan blue dye exclusion using a Neubauer chamber. Samples with more than 85% of live cells were used for the experiments.

### Histological Analysis

At different time points after PBS or Tp inoculation (3, 7, 12, 15, or 30 dpi), the ears and/or heart sections were fixed and preserved in 10% (v/v) formalin and then embedded in paraffin. Sections of 5 μm in thickness were stained with hematoxylin and eosin. In the heart sections, quantification of amastigote nests was estimated after the observation of at least 25 fields per preparation (set in triplicate with three preparations per condition) using ZEISS Primovert light microscope and Axiocam ERc 5s camera.

### Flow Cytometry

Single-cell suspensions from the skin, dLNs, and spleen were stained with a mix of monoclonal antibodies (Ab) conjugated with different fluorophores: anti-MHCII-PeCy5 (M5/114.15.2, eBiosciense), CD11c-FITC (N418) and CD11b-PE (M1/70) (both from BD Biosciences) or CD207-PEVIO770 (caa8-28H10), CD45-APC (ALI-4A2), CD3-biot (145-2C11), CD8-PerCP (53-6.7), and MHCII-FITC (M5/114.15.2) from Miltenyi Biotec. Staining with biotin-conjugated Ab was followed by PE-Cy7-streptavidin (BD Biosciences). Cells were acquired on FACSAria II flow cytometer (BD Biosciences) and analyzed by FlowJo 7.6 software. Gating strategies were used to exclude doublets (FSC-A *vs* FSC-H) and debris/dead cells by size and complexity.

### Parasite DNA in Tissues

Endpoint and quantitative (q)PCR were performed in order to detect parasite DNA in the skin and spleen. DNA samples were extracted using ADN puriprep T-kit (Inbio Highway, Argentina), a spin-column-based extraction protocol, following the instructions of the manufacturer. Individual samples were tested by endpoint PCR and qPCR with primers specific for *T. cruzi* genome satellite sequences (Duffy et al., 2009). *TNF* gen was used as internal control. The primer sets were as follows: *SAT* Fw (5′-GCAGTCGGCKGATCGTTTTCG-3′) and *SAT* Rv (5′-TTCAGRGTTGTTTGGTGTCCAGTG-3′) and *TNF* Fw (5′-GGTGCCTATGTCTCAGCCTCTT-3′) and *TNF* Rv (5′-GCCATAGAACTGATGAGAGGGAG-3′). Samples from infected and non-infected mice and without target DNA were assayed as positive and negative controls of amplification. Amplification was carried out as previously reported (Duffy et al., 2009; Davies et al., 2014; Poncini et al., 2015).

Quantitative PCR was performed using EvaGreen qPCR Mix Plus (Solis BioDyne) and was set in a StepOnePlus™ (Thermo Fisher Scientific). After 5 min of preincubation at 95°C, PCR amplification was carried out for 40 cycles (94°C for 10 s, 65°C for 10 s, and 72°C for 10 s). The plate was read at 72°C at the end of each cycle. Results were normalized to the internal control and expressed as arbitrary units (AU). Three independent experiments with three technical replicates per sample were conducted. Endpoint PCR was carried out as described by Davies et al. (2014). Briefly, the 20-µl reaction tube contained 100 ng of template DNA, 1 U of Taq (Pegasus, PB-L Productos BioLógicos, Argentina), 2 µl of buffer 10×, 1.5 mM of MgCl_2_, 0.2 µM of primers, 2 mM of dNTPs, and distilled water to complete the volume. The cycling condition was as follows: a first step at 95°C for 3 min followed by 35 cycles at 95°C for 45 s, 55°C for 60 s, and 72°C for 45 s and a final elongation at 72°C for 1 min. Amplification products were assessed by gel electrophoresis in 1% agarose TAE1X and 1× of Page GelRed (Biotium).

### Statistical Analysis

Student’s *t*-test was used for the comparison of two different experimental conditions and one-way ANOVA and Bonferroni’s or Dunnett’s post-test was used for multiple comparisons. Survival curve was analyzed by Kaplan–Meier. All tests were performed with GraphPad Prism^®^ 5.01 and a *p*-value <0.05 was considered significant.

## Results

### Inflammatory Foci at the Inoculation Site With bTp or mTp

Recently, we have reported differences in the *in vitro* stimulatory capacity and infectivity of mTp and bTp in the culture with DCs from different origins (Gutierrez et al., 2020). Here, we compare some immunologic parameters after id injection of both infective stages in the experimental model.

**Figure 1:**
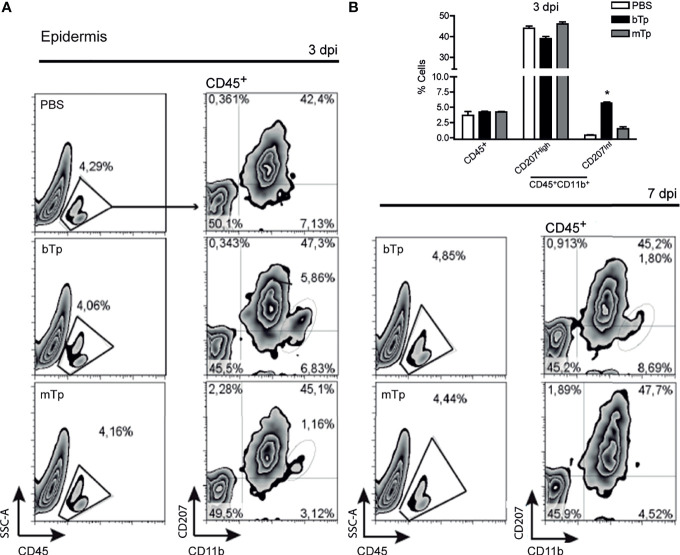
Metacyclic trypomastigote (mTp) or blood trypomastigote (bTp) inoculation recruits different leukocyte populations into the skin. **(A)** C3H/HeN mice were intradermally injected in the ears with bTp, mTp, or PBS (non-infected; NI). Gating strategy and characterization of leukocyte infiltrate in the epidermis at 3 days post-infection (dpi, left panels) and 7 dpi (right panel) analyzed by flow cytometry. One representative result of three independent experiments (three biological replicates with two pooled animals per condition) with similar results is shown. **(B)** Percentage of CD45^+^ and CD11b^+^CD207^+^ cells in skin samples at 3 dpi expressed as the mean + SEM. *p < 0.05 (*N* = 3, one-way ANOVA and Bonferroni’s post-test).

First, we analyzed the CD45^+^ leukocyte population in the ear skin at day 3 and 7 pi by flow cytometry (**Figure 1A**). No significant differences were detected in the percentage of CD45^+^ cells infiltrating the parasite entry site with bTp or mTp in comparison with PBS (**Figures 1A, B** **)**. Of note, at 3 dpi, bTp but not mTp displayed the presence of CD207^int^ cells (**Figures 1A, B** **)**, compatible with migratory monocyte-related myeloid cells as previously described for bTp (Poncini and González Cappa, 2017). At day 7 pi, a slight influx of leukocytes was detected at the infection site with both bTp and mTp, but no significant difference was found with PBS at the epidermal sheet of the skin (**Figure 1A**, right panels and data not shown). These results suggested that bTp and mTp would trigger different patterns of cell recruitment into the skin.

### mTp Displayed a Long Prepatent Period of Infection

Although previous works demonstrated a high sensitivity of bTp to gastric degradation (Cortez et al., 2012), controversially, other reports showed that mTp presented low infectivity in comparison with bTp, not only orally but also by different routes of infection (Dias et al., 2013; de Souza et al., 2014).

**Figure 2:**
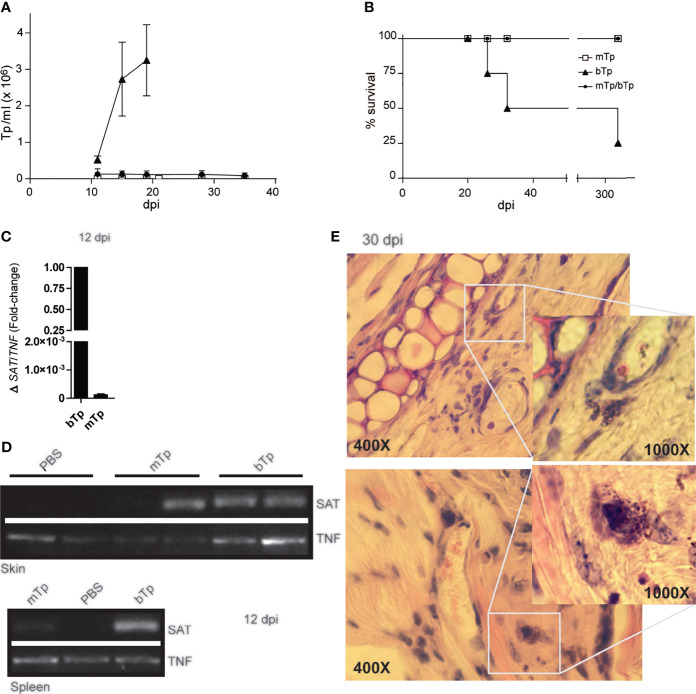
Animals injected with mTp present a prepatent infection. **(A)** Peripheral blood parasitemia and **(B)** mice survival were monitored during comparative experimental infections. Mice were inoculated with mTp (unfilled square), bTp (triangles), or mTp injected and challenged with an intraperitoneal lethal dose of bTp (filled circle) as described in the *Materials and Methods*. One experiment of five with four to five mice per group is shown. **(C)** Quantitative PCR from skin samples and **(D)** endpoint PCR from skin or spleen samples were conducted at 12 days post-infection. **(C)** Results were normalized to the internal control TNF and expressed as arbitrary units (AU). Three independent experiments with three technical replicates per sample were conducted. **(D)** SAT bands correspond to parasite DNA detection in ear skin and spleens in mice inoculated with mTp or bTp. TNF detection bands were also found in samples from PBS-injected control mice (non-infected; NI). One independent experiment of three with two technical replicates per condition is shown. **(E)** Skin sections obtained from mice at 30 dpi were stained with hematoxylin and eosin and analyzed by optic microscopy. Magnification of the boxes shows amastigote nests in dermal auditory pinna. Two representative images from different biological replicates are shown. Data are from three independent experiments set in duplicates with similar results. For microscopic examination, at least 25 fields were analyzed (five sections per animal). Original magnification: ×400 and ×1,000.

The infection with mTp displayed different presentation in oral *versus* gastric or by intraperitoneal inoculation of the parasite (Kuehn et al., 2014; Barreto de Albuquerque et al., 2015), and no reports have characterized the infection *via* the skin. Here, we observed that, in sharp difference with bTp, animals injected with mTp did not show detectable parasitemia, neither by fresh drop nor by microhematocrit during acute infection (data not shown and **Figure 2A**). In addition, animals injected with mTp survived the chronic infection (**Figure 2B**). We conducted PCR analysis in different tissues to test infection with mTp. DNA from the parasite was detected in the skin of mTp-infected mice by qPCR at a very low load in comparison with those infected with bTp at 12 dpi (**Figure 2C**). To discard contamination with parasite DNA at the inoculation site, we analyzed the infection by endpoint PCR in the skin and spleen at 12 dpi, confirming successful infection with both bTp and mTp (**Figure 2D**). Interestingly, at 30 dpi, parasite persistence in amastigote nests associated to skin infection was only detected in mice infected with bTp (**Figure 2E**). Next, we challenged animals infected with mTp with a lethal dose of bTp. Mice showed no detectable parasitemia during acute infection in addition to no mortality (**Figures 2A, B** **)**. These results suggested that the infection with culture-derived mTp generates a strong immunity against *T. cruzi*.

### Infection With mTp Enhances the Activation Status of DCs

APC impairments during *T. cruzi* infection were previously described by our group and others (Van Overtvelt et al., 1999; Alba Soto et al., 2003; Planelles et al., 2003; Poncini et al., 2008). Here, we analyze the activation status of myeloid (CD11c^+^CD11b^+^) and non-myeloid (CD11c^+^CD11b^−^) cells compatible with DCs in dLNs 2 dpi and spleens at day 15 pi with mTp and/or bTp.

**Figure 3:**
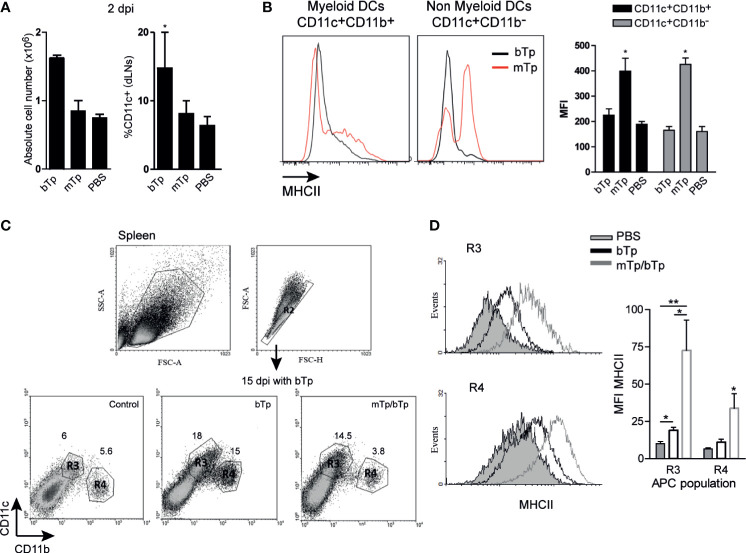
Analysis of DC populations from draining lymph nodes (dLNs) and spleen in mTp- and bTp-infected mice by flow cytometry. **(A)** Absolute cell number in dLNs (submandibular LN) expressed as the mean + SEM of three independent experiments (left) and percentage of DC population in total cells from dLNs (right). *p < 0.05 (one-way ANOVA and Dunnett’s post-test). **(B)** MHCII expression on myeloid and non-myeloid CD11c^+^ cells from animals infected with mTp (red line) or bTp (black line) in dLNs at 2 dpi (left). Mean fluorescence intensity (MFI) of MHCII expressed in myeloid and non-myeloid CD11c^+^ cells represented as the mean + SEM (right). Data are representative of three independent experiments (three biological replicates with duplicates per condition). *p < 0.05 (one-way ANOVA and Bonferroni’s post-test). **(C)** Gating strategy for the selection of myeloid and non-myeloid populations (CD11c^+^CD11b^+^, CD11c^+^CD11b^low/neg^, R1–4) in the spleen at 15 dpi. Data are representative of three independent experiments (three biological replicates with duplicates per treatment) with similar results. **(D)** MHCII expression in R3 and R4 populations. The gray histogram corresponds to NI samples, the gray line corresponds to the mTp, and the black line to samples from mTp-infected mice and challenged with bTp (left). Mean fluorescence intensity (MFI) of MHCII expressed in myeloid (R4) and non-myeloid (R3) populations represented as the mean + SEM (right). *p < 0.05, **p < 0.01 (*N* = 3 with duplicates as expressed above, one-way ANOVA and Bonferroni’s post-test).

As previously reported (Poncini et al., 2015; Poncini and González Cappa, 2017), bTp incremented the number of CD11c^+^ in dLNs at day 2 pi. However, neither cell recruitment nor the percentage of CD11c^+^ cells in dLNs from mTp-infected mice significantly differ from PBS-injected controls (**Figure 3A**). Interestingly, both the myeloid and non-myeloid CD11c^+^ populations incremented the expression of MHCII in mTp-infected animals compared with the ones injected with PBS or bTp (**Figure 3B**), suggesting a higher activation status in these cells. In the spleen, the CD11c^+^ population is enlarged in bTp-infected mice (Poncini et al., 2015; Poncini and González Cappa, 2017). Animals infected with mTp display no significant difference in CD11c^+^ cell recruitment to the spleen (data not shown). However, in mTp infection and challenged with bTp, mice sustained the recruitment of CD11c^+^CD11b^low/neg^ (R3) cells observed for bTp at 15 dpi (**Figure 3C**). Of note, both CD11c^+^CD11b^low/neg^ (R3) and CD11c^+^CD11b^+^ (R4) subpopulations in animals infected with mTp and challenged showed increased MHCII expression compared with bTp-infected mice, suggesting greater activation of DCs also in the spleen (**Figure 3D**).

### Mice Infected With mTp and Challenged With bTp Show Better Control of Parasite Load in Cardiac Tissue

To identify the extent of the results here described in cardiac tissue integrity, we analyzed the presence of amastigote nests in heart samples. Neither cellular infiltrates nor parasites were detected in animals infected with mTp. In fact, no differences were detected in the cardiac tissues between these samples and the negative control of the infection (data not shown).

**Figure 4:**
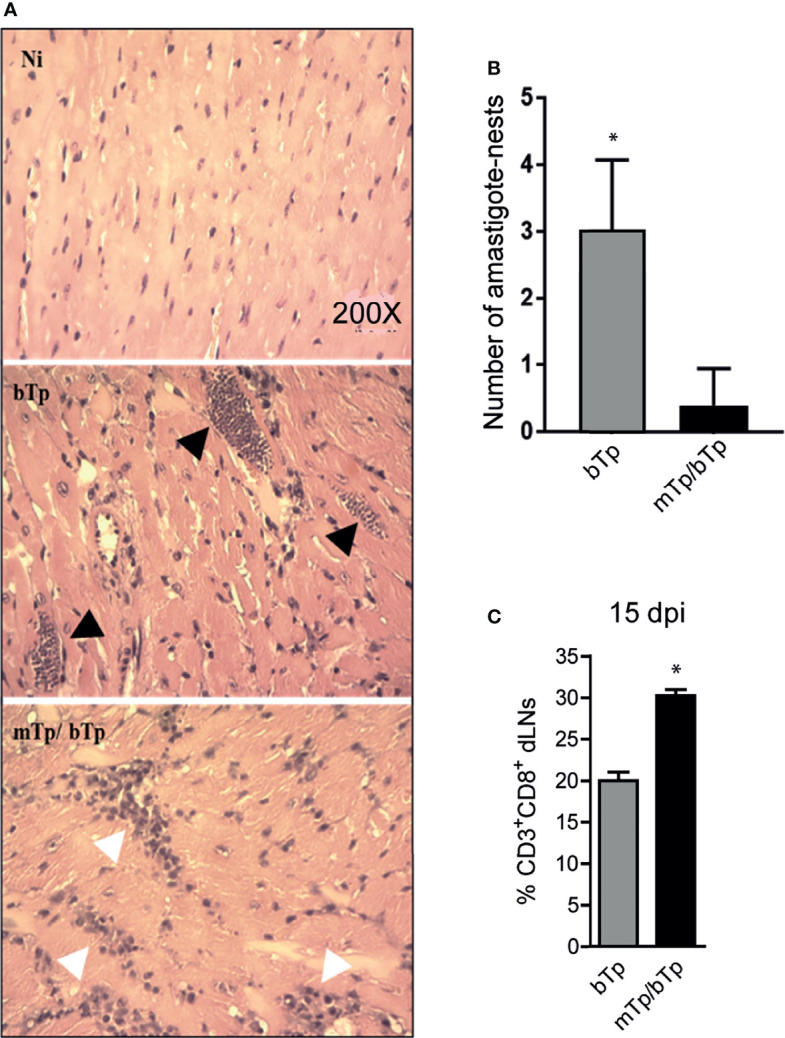
mTp infection enables better control of the parasite load in the cardiac muscle of mice. **(A)** Histology of cardiac tissue from NI, infected with mTp or bTp, or challenged animals, stained with H&E. Black arrows show amastigote nests and white arrows show cellular infiltrates in tissue. **(B)** Number of amastigote nests in cardiac muscle from 25 random fields per sample, *n* = 3, 15 dpi. *p < 0.05 (one-tailed, unpaired Student’s *t*-test). **(C)** Frequency of CD3^+^CD8^+^ T cells in dLNs at 15 dpi analyzed by flow cytometry. Bars represent the summary data of three independent experiments (*N* = 3 with duplicates per treatment; mean + SEM). *p < 0.05 (one-tailed, unpaired Student’s *t*-test).

Mice infected with mTp and challenged with bTp showed cellular infiltrates and less parasite load in cardiac tissue, measured as the number of amastigote nests as described in the *Materials and Methods* (**Figures 4A, B**). Consistent with the high activation status of APCs and the controlled parasitemia in animals infected with mTp and challenged with bTp, we detected an expansion of CD3^+^CD8^+^ T cells in dLNs at 15 dpi (**Figure 4C**). All results together strongly suggest that, while it is not sterilizing, the infection with *in-vitro*-obtained mTp induces the development of an effective immune response against *T. cruzi*.

## Discussion

Recently, it has been reported that DCs with diverse origins, bone-marrow-derived DCs in steady state, and epidermic-derived DCs responded differently to mTp and bTp infection and stimulation. In fact, not only the cell type but also the cellular activation status would condition APC response, in addition to the difference in the antigenic properties of the parasite (Gutierrez et al., 2020). As previously revised by Fernandes and Andrews (2012), trypomastigote-infective forms show big differences in molecular surface composition between mTp and bTp. In addition, the presence of lineages increments the variability since it was reported that there was a heterogeneous expression of surface molecules that trigger different signaling cascades during cell infection (Maeda et al., 2012; Mattos et al., 2014).

Studies in dogs demonstrated that mTp obtained from the vector and bTp developed dissimilar immune responses *in vivo* (de Souza et al., 2014). In addition, Dias and colleagues have demonstrated that mice inoculated with bTp orally or *via* the intraperitonal route present high parasitemias than those with mTp. However, they found that orally the infectivity could be influenced by the volume of the inoculum for mTp and the parasite load for bTp, variables that may ameliorate or counteract the acid gastric environment (Dias et al., 2013). These results reinforced the importance of revising the reproducibility and the scope of the models described in order to study *T. cruzi* infection. An increasing number of research studies demonstrate that parasite strains, genetic characteristics, routes of infection, and the host genetic background affect the immune response and the outcome of the infection (Planelles et al., 2003; Dias et al., 2013; Vorraro et al., 2014; da Costa et al., 2014; Barreto-de-Albuquerque et al., 2015).

Pathogen elimination or dissemination involves sequenced steps including its proper recognition by the host. Several studies have shown that resident macrophages are at the frontline as target cells for parasite invasion and multiplication (Cardoso et al., 2016), but *T. cruzi* can infect and persist in diverse cells and tissues (Barreto de Albuquerque et al., 2018; Ward et al., 2020). Here, we observed that intradermal injection of mTp or bTp resulted in polarized infections. Of note, since the very beginning of infection, mTp and bTp inoculated into the skin triggered a different cellular recruitment to the parasite portal of entry. Because of the lack of detectable parasitemia and the absence of mortality in mTp-injected animals, we performed parasite DNA detection by PCR at different tissues confirming infection despite detecting low parasite load. At first, we questioned the infectivity of mTp obtained *in vitro* from cultured epi, but other works using both mTp from *in vitro* metacyclogenesis or from insect feces demonstrated less virulence in the mTp infective stage independent of its origin (Dias et al., 2013; de Souza et al., 2014; Barreto-de-Albuquerque et al., 2015). Interestingly, at 30 dpi, amastigote nests in the skin were only detected during infection with bTp, demonstrating that depending on the infective stage, the skin can work as a tissue for the parasite establishment and persistence. For mTp, it was previously demonstrated that there was parasite persistence and replication in gastric mucosa (Hoft et al., 1996); however, there is scarce information for skin models of infection.

It has been already mentioned that immune response against the parasite can be modulated by the strain of the parasite, the stage, and the route of infection. Furthermore, recently, it was demonstrated that in vectorial transmission, feces contain a mix of epi and mTp in addition to intermediate stages. Adding complexity to the infection, Kessler and colleagues demonstrated infectivity by recently differentiated epi (Kessler et al., 2017). All these results open new clues about the role each parasite stage or other components such as the feces of triatomines or saliva play in the development of the immune response against the parasite (Mendes et al., 2016; Monteon et al., 2016).

Here, we observed that *via* intradermal inoculation, mTp are less virulent than bTp and that animals infected with mTp developed a long prepatent infection. In addition, the response developed by mice during mTp infection is strong enough to control a challenge infection with a lethal dose with bTp. Dias et al., 2013 found that although mTp infection presented low parasitemia, when orally inoculated, it displayed enhanced inflammatory foci in organs and tissues, probably associated with a potent antiparasite response. Consistent with this result, in the present work, animals showed enhanced activation of DCs in the dLNs and spleen, less parasite load, and enhanced cell infiltrate in cardiac tissues. All these data suggest that mTp are more immunogenic than bTp, partially confirmed here by the enlarged population of CD8^+^ T cells in dLNs from animals infected with mTp and challenged with bTp.

In conclusion, the results presented here strongly suggest that mTp and bTp trigger different early immune responses clearly evidenced in the skin model of infection. In addition, we confirm that mTp obtained *in vitro* successfully infected mice although with an extended prepatent infection, a situation that might mimic what is observed in *T. cruzi* vectorial transmission. The identification of the molecular pathways involved in early immune activation is an attractive strategy to identify possible targets for therapy development.

## Data Availability Statement

The raw data supporting the conclusions of this article will be made available by the authors, without undue reservation.

## Ethics Statement

The animal study was reviewed and approved by the University of Buenos Aires’s Institutional Committee for the Care and Use of Laboratory Animals (CICUAL).

## Author Contributions

BG, SMGC, and CP contributed to the concept and design of the study. BG and CP performed the experiments and analyzed the data. EL conducted the epi cultures. BG and CP wrote sections of the manuscript. All authors contributed to the article and approved the submitted version.

## Funding

This work was supported by Consejo Nacional de Investigaciones Científicas y Técnicas (CONICET), Fundación Bunge & Born, and Universidad de Buenos Aires (UBACyT 2017 20020160100117BA), Argentina.

## Conflict of Interest

The authors declare that the research was conducted in the absence of any commercial or financial relationships that could be construed as a potential conflict of interest.

## Publisher’s Note

All claims expressed in this article are solely those of the authors and do not necessarily represent those of their affiliated organizations, or those of the publisher, the editors and the reviewers. Any product that may be evaluated in this article, or claim that may be made by its manufacturer, is not guaranteed or endorsed by the publisher.
